# Chromosome loops arising from intrachromosomal tethering of telomeres occur at high frequency in G1 (non-cycling) mitotic cells: Implications for telomere capture

**DOI:** 10.1186/1475-9268-3-3

**Published:** 2004-09-29

**Authors:** Art Daniel, Luke St Heaps

**Affiliations:** 1 Department of Cytogenetics, Western Sydney Genetics Program, The Children's Hospital at Westmead, NSW 2145, Australia

## Abstract

**Background:**

To investigate potential mechanisms for telomere capture the spatial arrangement of telomeres and chromosomes was examined in G1 (non-cycling) mitotic cells with diploid or triploid genomes. This was examined firstly by directly labelling the respective short arm (p) and long arm subtelomeres (q) with different fluorophores and probing cell preparations using a number of subtelomere probe pairs, those for chromosomes 1, 3, 4, 5, 6, 7, 9, 10, 12, 17, 18, and 20. In addition some interstitial probes (CEN15, PML and SNRPN on chromosome 15) and whole chromosome paint probes (e.g. WCP12) were jointly hybridised to investigate the co-localization of interphase chromosome domains and tethered subtelomeres. Cells were prepared by omitting exposure to colcemid and hypotonic treatments.

**Results:**

In these cells a specific interphase chromosome topology was detected. It was shown that the p and q telomeres of the each chromosome associate frequently (80% pairing) in an intrachromosomal manner, i.e. looped chromosomes with homologues usually widely spaced within the nucleus. This p-q tethering of the telomeres from the one chromosome was observed with large (chromosomes 3, 4, 5), medium sized (6, 7, 9, 10, 12), or small chromosomes (17, 18, 20). When triploid nuclei were probed there were three tetherings of p-q subtelomere signals representing the three widely separated looped chromosome homologues. The separate subtelomere pairings were shown to coincide with separate chromosome domains as defined by the WCP and interstitial probes. The 20% of apparently unpaired subtelomeric signals in diploid nuclei were partially documented to be pairings with the telomeres of other chromosomes.

**Conclusions:**

A topology for telomeres was detected where looped chromosome homologues were present at G1 interphase. These homologues were spatially arranged with respect to one-another independently of other chromosomes, i.e. there was no chromosome order on different sides of the cell nuclei and no segregation into haploid sets was detected. The normal function of this high frequency of intrachromosomal loops is unknown but a potential role is likely in the genesis of telomere captures whether of the intrachromosomal type or between non-homologues. This intrachromosomal tethering of telomeres cannot be related to telomeric or subtelomeric sequences since these are shared in varying degree with other chromosomes. In our view, these intrachromosomal telomeric tetherings with the resulting looped chromosomes arranged in a regular topology must be important to normal cell function since non-cycling cells in G1 are far from quiescent, are in fact metabolically active, and these cells represent the majority status since only a small proportion of cells are normally dividing.

## Background

In both plants and animals, during early meiosis in normal cells there is a clustering of all or most of the telomeres of the entire chromosome set to a single region on the nuclear membrane [1-3]. This meiotic looping of chromosomes with clustered ends has been termed the *bouquet arrangement *which appears synchronously with synapsis of bivalents. The reason why the telomeres attach to the nuclear membrane in meiosis is not dependent on the presence of normal numbers of TTAGGG repeats and, in fact, still occurs in late generation Terc-/- mice without detectable pantelomere repeats [5]. In plants the meiotic telomere clustering can be inhibited by colchicine [6] but a polarization still remains within the nuclei such that microtubules and nuclear pores are still arranged in a region that normally would face the telomere cluster on the opposite side of the nuclear membrane [3]. In mammals the bouquet arrangement seen at early meiosis occurs with some minor differences between males and females [4] but has disappeared in both by diplotene/dictyotene. For mitosis there is much less data on the position or possible associations of telomeres and subtelomeres. However, the spatial arrangement of chromosomes at mitotic interphase has been studied intensively [7,8] but there are few studies with data on the principles that dictate nuclear organization. Nagele *et al. *[9,10] using whole chromosome paints on fixed normal diploid human cells described a radial array (rosette) of prometaphase chromosomes where the chromosomes were apparently arranged in two tandemly linked haploid sets. That interphase chromatin formed ring-like shapes was already known [11,12] but Nagele *et al. *[9,10] proposed that there was a chromosome order in each of the haploid sets in diploid cells during mitosis which was thought to be reversed with respect to one-another. A chromosome order was also described as being present at interphase in non-cycling cells [13] where the nuclear organization seems to be fundamentally different from that in dividing cells [14,15]. From observations in triploid cells, Nagele *et al. *[10] proposed that the three haploid sets were spatially arranged, two with the chromosomes arranged in tandem and the third with a reversed chromosome order. The relationship of subtelomeric regions to these concepts of a chromosome order within a radial chromosome array is less clear. Stout *et al*. [16] studied subtelomeric chromosome regions at interphase and showed that compared to interstitial chromosome sites, subtelomeres showed an increased number of somatic pairings. By FISH within living cells, Molenaar *et al. *[17] were able to demonstrate that these telomeric associations are dynamic. The rate of telomeric associations apparently depends on the stage of the cell cycle. Nagele *et al. *[18] utilising a telomere-specific peptide nucleic acid probe has demonstrated that the prevalence of such telomeric associations is far higher at interphase in non-cycling cells than in their cycling counterparts. In the present study we examine the telomere associations in mitotic interphase in human non-cycling cells of diploid or triploid karyotype. The cell types used were from skin, fetal cartilage, and long-term culture of chorionic villi but colchicine and hyptotonic treatments were avoided during cell harvest because of the potential effect of disrupting any topology present [6,19]. We report a new finding, the detection of looped chromosomes in mitotic G1 by the intrachromosomal tethering of short-arm (p) and long-arm (q) telomeres. This new finding has implications for the understanding of the normal dynamics of chromosome behaviour at interphase but also for the processes involved in telomere capture.

## Results

**Figure 1:**
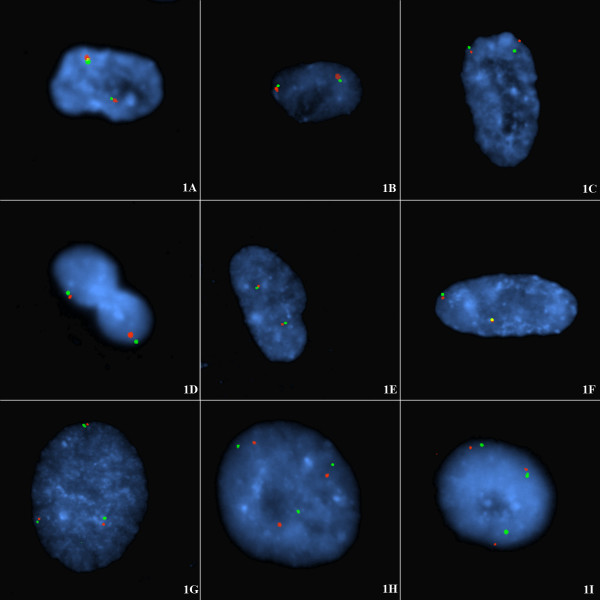
**A-I **Intrachromosomal tethering of the subtelomeres of each single homologue in diploid and triploid non-cycling interphase nuclei at G1. FISH of diploid (A-F) or triploid interphase nuclei (G-I) from the following cell lines: CG04-0743BBRS (diploid) derived from skin and CG01-2042YA (triploid) derived from CVS. These non-cycling cells were probed with p-subtelomeric probe (labelled with spectrum orange) and q-subtelomeric probe (spectrum green) for (A) chromosome 4; (B) chromosome 5; (C) chromosome 7, (D) chromosome 10, (E) chromosome 17, (F) chromosome 20, (G) chromosome 18, (H) chromosome 12, (I) chromosome 6. The proportion of p-q tethered signals is shown in Table 2. In each case the majority of cells (76%–85%) showed pairing of short-arm and long-arm subtelomeres from single homologues often arranged on opposite sides of the interphase nucleus. Note also that an interphase topology is exhibited such that oval rosettes of chromatin can be seen in the present study in figs 1H, 1I, an elongated rosette in fig 1C, and off-centre rosettes in figs 1A, 1B.

Fluorescence in situ hybridization (FISH) performed with the various subtelomere probes (Table 1) gave discrete signals in all experiments attempted. Figure 1 shows FISH of cells probed with the p-subtelomeres labeled red and q-subtelomeres labeled green for chromosomes 4, 5, 7, 10, 17, and 20, arranged respectively in figure 1A,1B,1C,1D,1E,1F (diploid cell line CG04-0743BBRS) and for chromosomes 18, 12 and 6 in figure 1G,1H,1I (triploid cell line CG01-2042YA). The proportion of p-q associated signals is shown in Table 2. The frequency of p-q subtelomere tethering ranged from 76–85% in the diploid cells but was a little less in the triploid cells (58–94%). Not all signal pairs were tethered. The percentage of diploid cells with *all *p-q signals tethered was 46–72% as compared to 33–58% in the triploid cells. This would be expected with more opportunity for interhomologous tethering in the triploid nuclei with an extra chromosome.

**Table 1:** Origin and derivation of the telomere clones used in the study.

**Clone**	**Chrom**	**Supplier**
**1186B18**	3p	Flint
**196F04**	3q	Incyte
**36P21**	4p	Incyte
**963K6**	4q	Flint
**189N21**	5p	Incyte
**240G13**	5q	Incyte
**62I11**	6p	Incyte
**57H24**	6q	Incyte
**164D18**	7p	Incyte
**3K23**	7q	Incyte
**43N06**	9p	Incyte
**112N13**	9q	Incyte
**306F07**	10p	Incyte
**137E24**	10q	Incyte
**496A11**	12p	Flint
**221K18**	12q	Incyte
**2111b1**	17p	ATCC
**362K4**	17q	Flint
**52M11**	18p	ATCC
**964M9**	18q	Flint
**1061L1**	20p	Flint
**81F12**	20q	Incyte

**Note: **The host strain was E. coli DH10B with kanamycin resistance in all cases except the clone 2111b1 (17p probe) which was ampicillin resistant.

**Table 2:** Rate of tethering in non-cycling cells at G1 interphase of p (short arm) to q (long arm) subtelomeric signals in single homologues.

Chromosome	Genome of cells	Numbers and [Percentage] of p-q signal pairs** tethered (95% confidence limits)	Numbers and [Percentage] of cells with all p-q signals tethered# (95% confidence limits).
4	Diploid	50/60 [83] (71–91%)	10/14 [72] (42–92%)
5	Diploid	123/148 [83] (76–89%)	26/41 [63] (47–78%)
7	Diploid	86/113 [76] (67–84%)	26/45 [58] (42–72%)
9	Diploid	ND*	ND*
10	Diploid	92/108 [85] (77–91%)	24/35 [69] (51–83%)
17	Diploid	62/76 [82] (71–90%)	13/28 [46] (28–66%)
20	Diploid	72/90 [80] (70–88%)	14/24 [58] (37–78%)
3	Triploid	25/30 [83] (65–94%)	5/10 [50] (19–81%)
6	Triploid	49/61 [80] (68–89%)	12/22 [55] (32–76%)
12	Triploid	57/82 [70] (58–79%)	8/24 [33] (16–55%)
18	Triploid	65/85 [77] (66–85%)	12/28 [43] (25–63%)

ND-not determined. * Cross-hybridization between 9q and 18p prevented analysis.

** p-q subtelomere signal pairs scored, irrespective of cell numbers.

#Separate scoring of discrete cells only; i.e. diploid cells with clearcut tethering of both p (short-arm) to q (long-arm) subtelomere signal pairs and triploid cells with all three p-q subtelomere signal pairs tethered.

**Figure 2:**
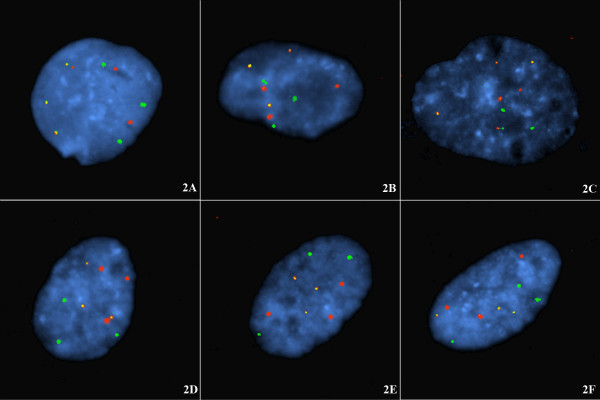
**A-F **Chromosome homologues at G1 in nuclei of non-cycling cells are spatially arranged without respect to non-homologues. Same cell line and same cell harvest as the triploid cells probed in Fig 1. Two combinations of three subtelomeric probes (see Table 1 for clones) are shown hybridized to triploid cells. In fig 2A-C the nuclei are probed with three single subtelomere probes from 4p (spectrum orange); 18q (spectrum green) and 6p (both spectrum orange and spectrum green labels, i.e. yellow signal). In figs 2D-F, the nuclei are probed with three subtelomere probes labelled 5p (spectrum orange), 12q (spectrum green), and 20p (spectrum orange and spectrum green, i.e. yellow signal). Note: There was no segregation into haploids sets of chromosomes at G1 interphase. Homologues were regularly arranged without any defined relationship to non-homologous signal groups; i.e. haploid sets of interphase chromosomes distributed to separate nuclear regions do not appear to exist. Note also there is a low frequency of isolated non-homologous associations: between 4p and 6p (Fig 2A); between 6p and 18q (Fig 2C), and between 5p and 20p (Fig 2D).

The triploid cells were used to test the likelihood that intrachromosomal pairing of subtelomeric signals was occurring rather than the pairing of p and q signals with the q and p signals of the other homologue(s). As can be seen in figure 1G,1H,1I, there were three p-q tethered signal pairs in the triploid interphase nuclei. Figure 2 shows single arm subtelomeric probes from three different chromosomes demonstrating that there is no linkage of positioning (chromosome order) between non-homologues. This is evidence challenging the claims of haploid groups being present at the interphase of non-cycling cells.

**Figure 3:**
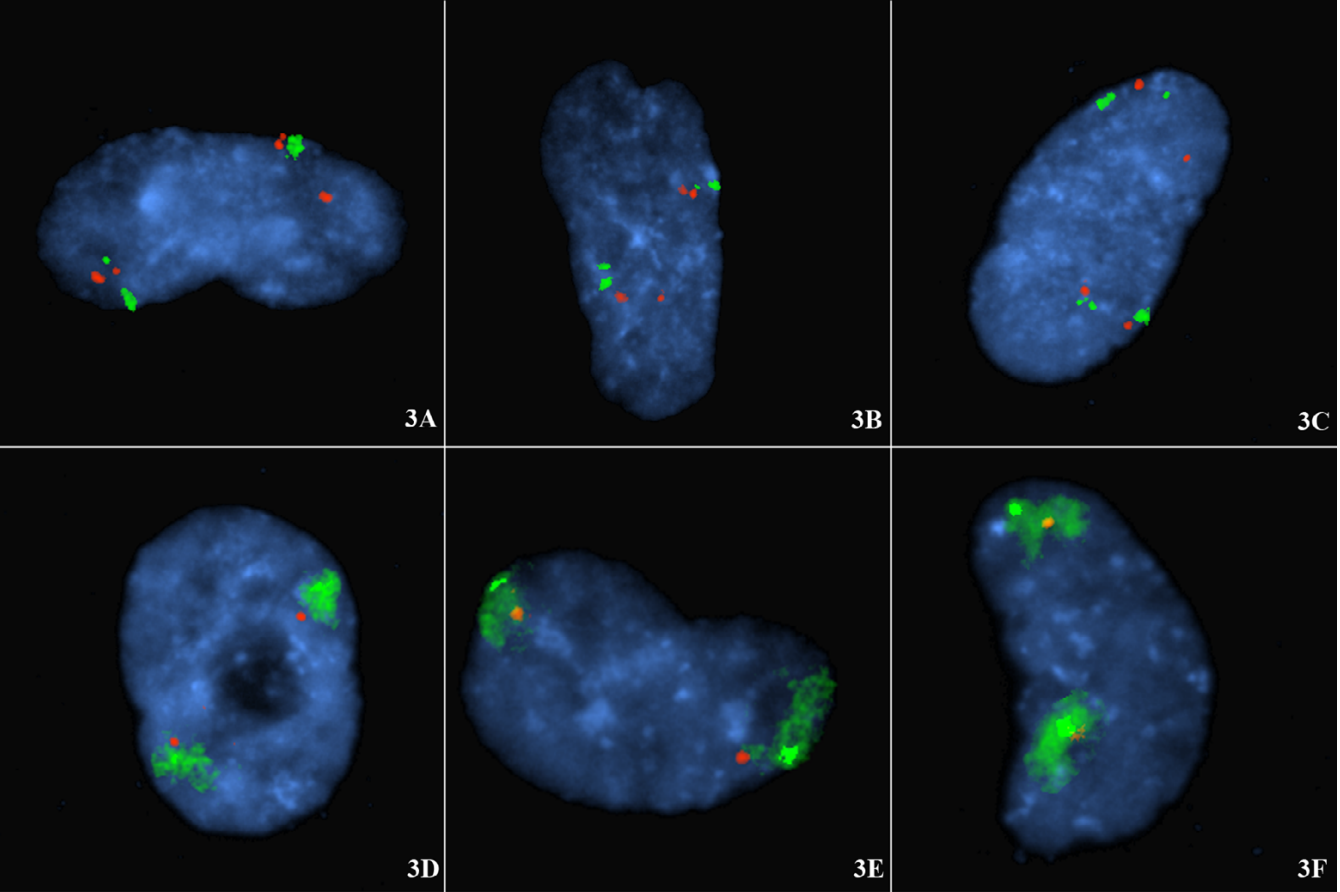
**A-F **Looped chromosomes in G1 arrested cells: the distribution of tethered subtelomeric signals coincides with chromosome domains. Diploid non-cycling cells harvested after confluence arrest. The diploid cells are from the same cell line as in Fig 1 (i.e. CG04-0743BBRS). Fig 3A-3C shows diploid cells probed for chromosome 15 with CEN15 (larger signal spectrum green); SNRPN at 15q12 (spectrum orange); PML at 15q22 (spectrum orange); and subtelomeric 15q probe (smaller signal spectrum green). Note: The two chromosome 15 domains coincide with and envelop the 15q subtelomeric signals (there is no currently recognized specific 15p subtelomeric sequence and hence no 15p subtelomeric probe). Fig 3D-3F shows diploid cells probed for chromosome 12 with the subtelomeric probes for 12p (labeled with spectrum orange) and 12q (labeled with spectrum green) and the WCP chromosome 12 (the spectrum green smear). Note: The three chromosome 12 domains as defined by the (directly labeled) WCP12 envelop the three tethered subtelomeric probe pairs. This confirms that the telomeric tethering represents looped chromosomes.

Further confirmation that the p-q tetherings in figure 1 were from single chromosomes is shown in figure 3. Figure 3A,3B,3C, shows chromosome 15 interstitial loci (diploid cell line CG04-0743BBRS) probed together with the 15 alpha centromeric probe, and a 15q subtelomeric locus. Separate chromosome domains surround the subtelomeric signals (Fig 3A,3B,3C). Similarly for chromosome 12 (Fig 3D,3E,3F), using the same diploid cells (CG04-0743BBRS), subtelomeric probe pairs are defined to occur within the two separate chromosome domains by jointly using WCP12.

## Discussion

### Evidence for short-arm and long-arm subtelomeres of the one homologue associating

This study shows that the pairings of red/green signals from the subtelomeres of the short-arm and long-arm respectively occur at high frequency in these non-cycling diploid nuclei. In many cases the association is so close that the subtelomeric signals are superimposed (e.g. figure 1A,1F). The pairs of red/green, p/q signals are from a single chromosome with the two diploid homologues arranged on different sides of the nucleus. This has been shown in this study in several ways. Firstly, it is highly likely that separate looped chromosomes are involved since the paired subtelomeric signals occur with small chromosomes (chromosome 17, 18, 20), intermediate chromosomes (7, 9, 10, 12) or large chromosomes (3, 4, 5) and are observed in triploid as well as diploid cells. Indeed the wide separation of the two subtelomeric signals from pairs of homologues (e.g. fig 1B,1D) supports the present interpretation that the telomeric tetherings of p-q signal pairs are intrachromosomal and not between homologues. Secondly, when interstitially located probes are used, for example on chromosome 15 (Fig 3A,3B,3C) in diploid cells, two distinct chromosome domains are seen. Thirdly, when subtelomeric probe pairs are used with a WCP probe for example on chromosome 12 (Fig 3D,3E,3F) in diploid cells, two distinct chromosome domains are seen that envelop the two tethered pairs of subtelomeric regions.

In diploid nuclei the pairs of tethered subtelomeric signals are distributed to two areas and in triploid nuclei (fig. 1G,1H,1I), the tethered signals are distributed to three areas. If the signal pairings were between the short-arm from one homologue with the long-arm of another it is especially unlikely in the triploid cells that the chromosomes could span the diameter of the nucleus consistently. This is especially unlikely in light of the finding by Nagele *et al. *[10] that the nucleus normally exhibits a rosette of (chromosome rich) chromatin with a less dense central core (doughnut shape). If inter-homologous telomeric associations were the explanation for the regular p-q signal pairings then, especially in triploid cells, chromosomal threads would have to be arranged in very complex formations across the chromatin poor cores of rosettes. Finally, there is separate evidence that there are small non-overlapping chromosome territories at interphase in mammalian cells [20,21] where the chromosomes are extended but not entwined. In the present study we have also been able to show the presence of these interphase chromosome domains both with the use of several probes spanning the length of chromosomes (e.g. Fig 3A,3B,3C) or with chromosome paints (e.g. Fig 3D,3E,3F).

Nagele *et al. *[18] showed that there were very few coincident telomeric associations (TA's) in rapidly cycling mitotic cells. However, these authors showed [18] that in non-cycling cells there was a high rate of double associations, and a lesser frequency of triple and quadruple associations or unassociated telomeres. These authors [18] concluded that the replicative status of the cells was the prime determinant in the level of telomere associations. The finding of a high intrachromosomal p-q telomere association rate in the present study probably explains the underlying high telomere association rate described by Nagele *et al. *[18]. In that study [18], a universal telomere probe was used so the specificity of the associations, if present, was unrecognisable. In the present study, there was a high (~80% but not saturated) rate of intrachromosomal pairing with only ~20% of telomeres unpaired with their homologous subtelomere. These two studies can be reconciled if the apparently (~20%) unpaired subtelomeres (present study) were actually associated with non-homologous subtelomeres. Fig 2 shows the presence of an underlying low rate of non-homologous telomere tetherings in these G1 arrested cells.

### Regulation of telomere associations

In early meiotic cells the presence of the normal numbers of universal TTAGGG sequences is not required for massed telomere clustering [5]. A complementary finding was reported by Nagele *et al*. [18] who showed that in late passage mitotic cells the number of telomere associations (TA's) did not increase during progression to late passage crisis. This indicates that telomere shortening did not increase the number of TA's. Since the pantelomeric repeats occur at all telomeres, the specific intrachromosomal association presently observed also cannot be due to their presence. Neither can the mechanism of tethering be related to chromosome specific subtelomeric sequences since the two homologues with identical sequences remain separated (Fig 1).

There clearly are similarities between the looping of chromosomes seen in the present non-cycling mitotic cells and in the chromosome bouquets of early meiosis [2,3]. These two apparently disparate phenomena may be related. If the synapsis of bivalents, unnecessary in mitotic cells, was removed from the meiotic bouquet arrangement mechanism, the intrachromosomal tethering of separated homologues as presently observed is what may be left. This mitotic looping may have been originally present since meiosis is believed to have evolved from mitosis.

### Chromosome topology at interphase

The global organisation of the interphase nucleus has provoked the interests of cell biologists for several decades but detecting the presence of any macromolecular domains has been challenging [8]. Nagele *et al. *[9,10] was able to confirm with Feulgen staining and FISH that the chromosomes were arranged in rosettes, a ring of chromatin with partly-condensed chromosomes, which persisted through mitosis and was even maintained in the daughter cells at telophase. Oval rosettes can be seen in the present study in figs 1H,1H, and 3D; an elongated rosette in fig 1C, and off-centre rosettes in figs 1A,1B, and 2B. Through the use of FISH with chromosome specific alphoid probes and whole chromosome paints, Nagele *et al. *[10,13] attempted to show that chromosomes in the rosettes appeared to be in an orderly arrangement in both diploid and triploid cells. These authors interpreted this order as specifically positioned haploid sets [9,10,13]. The pairing of subtelomere signals in non-cycling cells at interphase, as in the present study, is in some aspects consistent with these prior observations though we do not accept that haploid sets are spatially segregated and we found no evidence for an interphase chromosome order in the non-cycling cells of our cell lines. We have repeated this work with centromeric probes (not shown) and again there was no evidence of haploid groups or of a regular chromosome order though widely spaced homologous centromeric signals are usually observed (with respect to each chromosome considered separately).

With respect to telomeric tethering in cycling cells (at G2) no such p-q telomeric tethering pattern is present in our observations of lymphocytes (not shown) and the only associations are of sister chromatids. That most lymphocytes are at G2 can be observed by the doubled signals representing sister chromatids (not shown) which is in contrast to the single (chromatid) signals in the unreplicated G1 nuclei (see fig 1).

With centromeric and painting probes, Nagele *et al. *[9,10,13] detected the presence of what they believed to be haploid sets of chromosomes in both diploid and triploid cells with the sets on opposite sides of the nucleus. In some cell shapes (e.g. elongated, polymorphic, or lenticular shaped cells) this regular order was obscured but in spherical nuclei it was mostly evident. Whereas there is often a spatial separation of the telomeric signals from the various homologues of the diploid or triploid G1-arrested cells in the present data (see fig 1) there was no evidence for a chromosome order or haploid groups in the cell nuclei (fig 2). In the explanation of Nagele *et al. *[13] the haploid sets these authors proposed represented maternal and paternal chromosome contributions. In the present data each set of identical homologues (two in diploid or three in triploid cells) appear to be arranged without respect to those of other chromosomes (fig 2), i.e. the spatial arrangement is not an interchromosomal phenomenon. This means the theoretical haploid sets of chromosomes described by Nagele *et al. *[9,10,13] probably do not exist. Figure 2 illustrates the two experiments performed in the current study to address the possible existence of haploid sets. These comprised examining the chromosome order for the single telomeres 4p (labelled with spectrum orange – Vysis, Downers Grove, Illinois), 18q (spectrum green), and 6p (spectrum green and spectrum orange, i.e. yellow signal) jointly hybridised to the same confluence arrested cells, and in a second experiment: 5p (spectrum orange label), 12q (spectrum green), and 20p (spectrum green and spectrum orange) hybridised to a second slide of triploid cell nuclei. These cells are from the same harvest as those shown to display the interphase topology of p-q intrachromosomal subtelomere tethering. In these latter results, homologous subtelomeres were regularly arranged without any defined relationship to non-homologous signal groups. This demonstrates that there is no interchromosomal order transferable between nuclei and challenges the concept of the presence of haploid sets within these non-cycling cells.

In the view of Nagele *et al. *[10] the dual odd topology that he observed with (i) homologues arranged on opposite sides of the nuclei (diploid cells) or regularly arranged around the nucleus (triploid cells), and (ii) a chromosome order possibly manifesting as "haploid sets" may just be a relic of fertilization. Whereas, in our view, these intrachromosomal telomeric tetherings with the resulting looped chromatids must be important to normal cell function.

### Possible relationship of telomere tetherings to telomere captures

As reviewed by Ballif *et al. *[23] there are two general pathways whereby chromosomes can acquire a new telomere and thus become stabilised. Firstly, by "telomere healing", i.e. the direct addition of telomeric repeats by: (i) telomerase [24] or by (ii) telomerase-independent recombination-based mechanisms [reviewed in [25]]. The second pathway is by "telomere capture" in which a chromosome acquires a telomere from another chromosome or chromosome end [reviewed in [23]]. Telomere captures are observed in two forms, those that are within the one homologue or intrachromosomal telomeric captures or transpositions [22,23], and those between non-homologues [26]. Ballif *et al. *[23] considered four different models for telomeric captures involving the p and q arms of a single homologue (intrachromosomal captures). These telomeric captures where the telomere from one chromosome arm is deleted and replaced by a telomere from the other arm of the homologous chromosome were termed *intrachromosomal transpositions of telomeres *[22] because of the uncertainty that simple reciprocal translocation was involved in this type of telomere capture. Ballif *et al*. [23] suggested that *breakage induced replication *(BIR), reviewed in Kolodner *et al. *[28], was the most likely model for these intrachromosomal captures based on their observation that there was observed heterozygosity between the duplicated ends on the one chromosome. This mechanism was initially described by Reddel *et al. *[27] under the unwieldy name "alternative lengthening of telomeres mechanism". Ballif *et al. *[23] suggested that BIR simply copied the sequence from the other end of the same homologue. Furthermore, that obligatory crossing-over during meiosis would mean that heterozygosity between duplicated ends would occur in many cases. The detection in the present study for the first time that in non-cycling mitotic cells in G1 most short-arm and long-arm telomeres from the one chromosome are tethered together is a likely staging point for mitotic events as per the fourth model of telomere capture reviewed in Ballif *et al. *[23]. This fourth model is that of the present authors in a prior study [22]. In the explanation of that fourth model by Ballif *et al*. [23], telomere capture was believed to occur by a pre-meiotic interhomologous exchange. The imbalanced chromosome was then generated through normal meiotic recombination. This (model) thus resulted firstly in a balanced translocation, termed *telomere transposition *by Daniel *et al. *[22] since *reciprocal *translocation was unproven. This translocation relocated the telomeres to the opposite chromosome arm and then by recombination the result was a duplication of one telomere on both chromosome ends and a deletion of the other. For this model to be correct a high frequency of balanced telomeric translocations would have to occur. These would be observed as large pericentric inversions and are rarely reported – see review in Daniel, 1988 [30]. However, the transposition of telomeres to opposite chromosome ends resulting in large pericentric inversions would not be easily noticed during FISH in many cases. This is in contrast to translocations between non-homologues which are very obvious to an observer in a FISH study. In this connection, for telomere translocations between non-homologous the rate of clinically ascertained balanced translocations has been reported as very high. Flint and Knight [26] record that for the several types of (non-homologous) telomeric rearrangements: unbalanced translocations account for 54% of cases; deletions for 39%; and duplications for 6%. According to Flint and Knight [26] in almost all cases unbalanced translocations occur because a parent carries the balanced form. When the data used to draw this conclusion are scrutinised, see De Vries *et al. *[29] it includes many rearrangements that are microscopically detectable, i.e. essentially regular reciprocal translocations. Such latter rearrangements are not really "telomere captures", are often familial, and would be expected to be associated with a high rate of balanced carriers. In our experience (Greg Peters and Luke St Heaps – CHW Telomere Study Group) we have not detected a balanced carrier of a telomere capture of either the intrachromosomal type or the interchromosomal type. In our view the issue of the frequency of balanced telomere rarrangements needs to be revisited since telomere captures are technically sub-microscopic telomere rearrangements. This data impinges on the likelihood that BIR is the preferred method of telomere capture [see that view expressed in ref [23]]. Since with the BIR model immediate recombinants are formed with no balanced carriers, if balanced (telomere capture) carriers are frequently reported, then BIR is ruled out as the common mechanism of telomere capture. This judgement currently cannot be performed without a more rigorous approach to the whole data set and additional assessment of the de novo or alternative origin of telomere rearrangements.

## Conclusions

A topology for telomeres was detected where looped chromosomes were present at G1 interphase in confluence arrested cells. It was shown that the p and q telomeres of each chromosome in G1 cells associate frequently (80% pairing) in an intrachromosomal manner which was confirmed by studying chromosome domains with interstitial probes (chromosome arms) and WCP probes. It was found that homologues were regularly arranged without any defined relationship to non-homologous signal groups; i.e. there was no apparent chromosome order on different sides of the nuclei and no segregation into haploid chromosome sets was detected. The normal function of this high frequency of intrachromosomal telomeric pairings is unknown but a potential role is likely in the genesis of telomere captures whether of the intrachromosomal type or between non-homologues. In our view, these intrachromosomal telomeric tetherings with the resulting looped chromosomes arranged in a regular topology must be important to normal cell function since non-cycling cells in G1 are far from quiescent, are in fact metabolically active, and these cells represent the majority status since only a small proportion of cells are normally dividing.

## Materials and methods

### Cell culture

Cell lines were retrieved from liquid nitrogen, washed in Dulbecco's phosphate buffer (DPB) and reconstituted in Hams F10 medium. The following lines were used: a diploid skin fibroblast line CG04-0743BBRS with karyotype 46,XX derived from fetal cartilage and a triploid 69,XXX cell line CG01-2042YA of diandric origin derived from mesodermal cells of a chorionic villus biopsy. These were cultured until they reached confluence via contact inhibition. At this stage the cells exhibit a number of swirls of closely packed cells in parallel. They were then severally prepared for trypsin harvest usually 48 hours after the last media change without colcemid/colchicine treatment and without the usual 0.075 M KCl hypotonic treatment. The cells were trypsinised off and fixed three times in 3:1 methanol to glacial acetic acid and were stored at room temperature (R.T.) in fixative for 1–3 days. This period allowed some mild acidic digestion of the chromatin and spreading of the nuclei when slides were prepared. At the end of the storage period, cells were rewashed once with fresh fixative and dropped onto glass slides as per routine techniques, and stored on trays in a low humidity cabinet until used for FISH.

### Choice of probe and probe label

The identity of the probes used in the study is shown in Table 1. The clones containing the DNA for the subtelomere probes were obtained from three sources: Incyte Genomics (Fremont, California); Dr Jonathan Flint (John Radcliffe Hospital, Oxford, U.K.), via Dr David Mowat, or the ATCC, (Manassas, Virginia). All were grown in Luria Broth (LB) with kanamycin by standard techniques unless specified otherwise (Table 1). Plasmid DNA was extracted with QIAGEN midi kits as per the manufacturer's instructions except that DNA elution was achieved at 60°C overnight. Probes were all labelled by nick translation (using VYSIS kit and the fluorophores spectrum orange and spectrum green, Vysis, Downers Grove, Illinois) as per the manufacturer's instructions.

### Fluorescence in situ hybridization (FISH)

Slides were pretreated with a combined Pepsin/Rnase step. This was performed by prewarming RNAse and pepsin to 37°C, 200 μl of RNAse (0.1 mg in saline/sodium citrate – 2xSSC) was dispensed onto each slide, coverslipped and incubated at 37°C for 40 minutes in a humidified chamber. Coverslips were removed and slides washed twice for 5 minutes in 2xSSC at room temperature (RT). Slides were briefly drained and 200 μl of pepsin (0.2% in 0.01 M HCl) was placed on the slides, coverslipped and incubated at 37°C for 30 seconds. Coverslips were removed and slides were washed twice for five minutes in phosphate buffered saline (PBS) at RT. Fixation was with 6% paraformaldehyde in PBS, by dispensing 200 μl/slide, and adding a coverslip for 2 minutes at RT. Slides were then washed twice for five minutes in PBS at RT, dehydrated through 70, 90 and 100% ethanol for 3 minutes/wash at RT, and air dried. Probes in hybridisation mix were stored at -20°C, removed and thawed for 30 minutes; dispensed onto slides, covered with 15 mm diameter coverslips, and sealed with liquid rubber – art cement. Joint denaturation was achieved at 75°C for 5 minutes on a Omnigene hot plate, transferred to a humidified hybridization chamber at 37°C and hybridised overnight. After this the coverslips were removed. Post-hybridization washes were 0.4 SSC/0.3% NP40 at 73°C for 2 minutes then quickly transferred to 2xSSC/0.1% NP40 at RT for 1 minute. Slides were counterstained in DAPI and then rinsed and air dried. When ready, slides were mounted in antifade (2.3% DABCO in 40% glycerol/0.02 M TRIS-HCl) and covered until fluorescence examination. Slides were examined on a Zeiss Axioscop 20 fitted with a Zeiss fluoarc light source and images captured on an Applied Imaging Cytovision station using the false colours that are attributed by the software.

### Scoring of signal pairings to detect telomere tethering

Initially, the subtelomere probes were labelled in Spectrum Orange for all short arms and Spectrum Green for all chromosome long arms. Cells were separately probed with the two subtelomeric probes for a single chromosome at the one time. Probe pairs were used for the subtelomeres of chromosomes 1, 3, 4, 5, 6, 7, 9, 10, 12, 17, 18, and 20. Images were captured for a large number of cell groups for each chromosome and pairings were scored on the captured images. Signals were interpreted as paired if the distance between signals was 10% or less of the greatest diameter of the nucleus (many cells were oval in shape). In addition to the above subtelomeric probe pairs, other probes were used to investigate the frequency of non-homologous tetherings (subtelomeric probes 1p and 9q, see Table 3) and the coincidence of the subtelomere tetherings and interphase chromosome domains (Fig 3). For the latter experiment the following Vysis probes were used: PML (promyelocytic leukemia locus) mapping to 15q22, SNRPN (small nuclear ribosomal protein locus) mapping to 15q12 – both labelled with spectrum orange; CEN15 (a probe for alpha centromeric sequences specific to chromosome 15) labelled with spectrum green. In addition, the chromosome 12 subtelomeric probes, i.e. 12p (labelled with spectrum orange) and 12q (spectrum green), and the WCP (Vysis whole chromosome painting probe) for chromosome 12 (spectrum green).

**Table 3:** Rate of subtelomeric tetherings of non-homologues in G1 of non-cycling cells.

Pairs of telomeres tested for tethering	No (%) of signal pairs tethered	95% confidence limits
1p telomere; 9q telomere*	7/109 (6.4)	2.6–12.8%

Note: This is a control for chromosome specific p-q subtelomere signal pair tethering (Fig 2, Table 2). For this experiment the 1p subtelomere was labelled with spectrum orange and the 9q subtelomere with spectrum green. Subtelomere tethering between these non-homologues was not increased over chance expectation. The telomere pair* 1p and 9q were chosen because they represent one of the most frequent telomere translocations reported (Lisa Shaffer, personal communication 2004). Up to 10% of signal associations in a two colour matrix can be regarded as random (VYSIS guidelines for interphase FISH scoring). There was no departure from randomness for tethering with respect to these two pairs of non-homologous subtelomeres.

### Additional experiments to detect a regular chromosome order reflecting the possible existence of haploid sets regularly arranged around the nuclei

Two such experiments were performed in the current study (see fig 2). These comprised examining the chromosome order for the single subtelomeres (see Table 1 for clones) 4p (labelled in spectrum orange), 18q (spectrum green), and 6p (spectrum green and spectrum orange, i.e. yellow signal) jointly hybridised to the same triploid cells. In a second experiment subtelomeres were labelled as follows: 5p (spectrum orange), 12q (spectrum green), and 20p (spectrum green and spectrum orange, i.e. yellow signal) hybridised to a second slide of diploid/triploid cell nuclei.

## Authors' contributions

AD designed the study, captured and analysed all FISH signals, and drafted the manuscript.

LH performed all growing of probes, labelling of probes, and most probe hybridizations.
